# Communicating abnormal cervical cancer screening results – a focus group study with general practitioners in Norway

**DOI:** 10.1080/02813432.2025.2597785

**Published:** 2025-12-05

**Authors:** Ingvild Brenna, Bente Prytz Mjølstad, Ingrid Baasland, Heidi Gilstad, Marit Solbjør

**Affiliations:** ^a^ Department of Public Health and Nursing, Faculty of Medicine and Health Science, Norwegian University of Science and Technology, NTNU, Trondheim, Norway; ^b^ Department of Public Health and Nursing, Faculty of Medicine and Health Sciences, General Practice Research Unit, Norwegian University of Science and Technology, NTNU, Trondheim, Norway; ^c^ Department of Cervical Screen Norway, Cancer Registry of Norway, Norwegian Institute of Public Health, Oslo, Norway; ^d^ Center for Academic and Professional Communication (SEKOM), Department of Language and Literature, Faculty of Humanities, Norwegian University of Science and Technology, NTNU, Trondheim, Norway

**Keywords:** General practitioner, cervical cancer screening, HPV, health literacy, communication, shared decision making, qualitative research

## Abstract

**Background:**

A gradual transition from cytology-based screening to Human Papillomavirus (HPV) testing within the cervical cancer screening program has resulted in new routines for follow-up, and new challenges for communication of abnormal test results. General practitioners (GPs) have an important role in the screening program, as they are the primary performers of the screening test, they communicate test results to patients and refer them to a specialist if necessary.

**Objective:**

The study explores what consequences the introduction of HPV testing in the cervical cancer screening programme has for GPs’ professional practice and communication with patients.

**Design, setting and subjects:**

Qualitative focus group study including 32 GPs in Central Norway who conduct screening tests within the cervical cancer screening programme.

**Results:**

The overall concern of the GPs was to communicate abnormal test results in a way that ensured appropriate follow-up, without causing unnecessary worry. Staying updated on revised screening guidelines and maintaining their role as medical experts when communicating results to patients could be challenging. GPs shared the responsibility for follow-up after an abnormal result between themselves, the women, and the screening programme. Reciprocal familiarity between GP and patient guided decisions about what and how to communicate, and how to balance the shared responsibility. GPs used their professional judgement to assess patients’ informational needs and tailored information accordingly.

**Conclusion:**

GPs manage the challenges of communicating abnormal screening results by sharing responsibility and using their professional judgement. Strengthening support and communication tools may enhance their role in the screening programme.

## Introduction

Persistent Human Papillomavirus (HPV) infections cause 99% of cervical cancers [1]. Improved testing technologies [2] has allowed Norway and other European countries to adopt HPV testing as the primary cervical cancer screening method [3]. In Norway, Cervical Screen Norway issues invitations to women aged 25–69 years. Organized cervical cancer screening is associated with a substantial reduction in incidence [4] and mortality from cervical cancer [5]. HPV testing in Norway and the high prevalence of HPV, has led to more abnormal test results [6]. Most HPV infections clear within a few years, but some high-risk HPV infections persist and may lead to cancer if left untreated [7]. This results in varied test outcomes and follow-up needs, creating diverse information needs amongst patients.

General practitioners (GPs) in Norway hold a unique position in cervical cancer screening, including communicating test results and follow-up recommendations. After the shift to primary HPV screening, many GPs were insecure about new guidelines [8]. Similar challenges were reported in Australia [9], especially regarding the change from a 3 to 5-year follow-up interval for patients with a negative HPV test [10]. GPs face difficulties balancing this task with other responsibilities, keeping updated on guidelines and addressing the emotionally charged nature of HPV [11]. Cervical screening results are communicated in many ways, both concerning content and information channel [12]. In Sweden, gynecologists often used telephone or letter to explain abnormal results, tailoring messages to reduce patient anxiety [13]. The main concern of Swedish health personnel was to reassure patients that the cervical cancer screening results did not indicate cancer, and they experienced that oral communication prevented anxiety amongst patients [14].

Many women find it challenging to understand the meaning and implications of the cervical cancer screening result [15]. After implementation of primary HPV screening in England, many women were unfamiliar with the possibility of receiving an HPV positive test result and needed more information to understand its meaning and implications [16]. Received information is not always in line with women’s needs [17] who often search the internet for answers after consultations about cervical cancer screening [18].

Around 30% of the Norwegian population have low health literacy [19]. Health literacy is defined as the ability to find, understand, and use information to promote good health [20]. In general, health personnel often overestimate their patients’ health literacy [21]. This can result in too advanced information to patients about cervical cancer screening [22]. Instead of using assessment tools, GPs often rely on knowing their patients and on intuition when assessing health literacy [23]. While intuition can lead to over- or underestimation of health literacy, knowing the patients over time can contribute to more accurate assessment [24]. This indicates that GPs face potentially complex communication when delivering cervical cancer screening results, potentially creating new demands for GPs’ professional practices.

## Theoretical framework: professionalism

Sociological theories on professionalism suggest that the roles and practices of medical professionals are changing in society [25]. Having acquired unique knowledge through formal education, physicians conduct their professional expertise to solve individual health problems and problems related to public health [25], while administering their resources in a fair and wise way [26]. GPs’ professional expertise includes conducting biomedical assessments, communicating and conveying results to patients, and being updated on professional, institutional, and national guidelines and regulations [27]. When norms and regulations change, GPs must adjust their practices accordingly. As professionals, physicians are entrusted with autonomy to choose actions within the restrictions of directives and rules guiding their practice. Their expertise puts them in position to make sound professional judgements and decisions through discretionary reasoning, which is to find well-justified answers to their tasks [26]. This also applies to communication of test results, from for instance HPV testing. The demand for communication skills among physicians has been growing as the relationship between physician and patient has evolved from a paternalistic model to one characterized by mutual respect and collaboration [28]. As the primary contact for a wide range of patients’ health issues, GPs are in position to establish doctor-patient relationships with the potential for mutual commitment and responsibility, based on GPs’ knowledge about the patient as a person [29].

These dynamics become particularly salient in the context of cervical cancer screening, where the shift from cytology to HPV-testing has introduced new types of test results and uncertainties. Patients may now require different kinds of information and reassurance, which could put new demands on GPs’ professional practices. This study investigates how GPs enact their professionalism when communicating abnormal cervical cancer screening results to their patients within the HPV-based cervical cancer screening programme in Norway.

## Materials and methods

### Study design

Our methodological choices are underpinned by social constructivism, which sees construction of knowledge as context dependent and created through social interaction. From this perspective knowledge is regarded as situated, with the researchers playing an active role in the production of knowledge [30]. We conducted a focus group study with GPs experienced in cervical cancer screening and follow-up of patients. In health research, focus groups have been widely used for exploring professional experiences [31]. Focus groups are well suited for investigating experiences, meanings and reasoning related to a variety of topics and to capture interaction and discussions within participants [32]. Such discussions can inform issues of what is seen as ordinary practices, how positions are constructed within a group and identify discrepancies in opinions [33]. Focus groups composed of participants who already know each other and share professional backgrounds may encourage open sharing and facilitate thoughtful discussion [32]. We recruited pre-existing Continued Medical Education (CME) groups, allowing GPs to report the focus group session as a documented CME meeting. Each focus group session was followed by a lecture on cervical cancer screening held by IBA, a specialist in gynecology. This design gave participants the opportunity to pose questions about cervical cancer screening and provided an educational element for the CME-meeting.

### Setting

Norway is a welfare state with universal health insurance for all citizens [34]. A GP scheme gives all citizens the right to a regular GP. On average, each GP has around 950 patients [35]. General practice is a specialty for medical doctors. To maintain their specialist status as a GP, participating in a CME group is mandatory, with a minimum of 20 h during a 5-years period [36]. Consultation fee is around 200 NOK (€20), and citizens can book an appointment with their GP according to their needs. The Cancer Registry of Norway administers the screening program and is responsible for sending reminders to eligible women, while women are responsible for booking an appointment with a screening provider, primarily their GP (Cancer Registry of Norway, 2023). Cervical Screen Norway has elaborated a flow chart with national guidelines for follow up of cervical screening results [37], showing the timeline for follow up of abnormal test results (Supplementary textbox 1). Once analyzed, test results are communicated from the laboratories to the GPs, along with recommendations for follow-up. GPs communicate results to the patient, usually in writing through *Helsenorge.no*, a digital platform for provider-patient communication. On their website, Cervical Screen Norway offers some examples of standardized information letters, but no checklist or guidelines for how to communicate test results. When test results indicate the need for a colposcopy or biopsy, GPs refer the patient to a gynaecologist.

### Recruitment

We used a combination of purposeful and convenience sampling to recruit participants who could provide information about the topic of interest. Inclusion criteria were having regular experience with performing cervical screening, and the willingness to participate in the focus group together with their CME group. Recruitment was done through two closed Facebook groups for GPs which has a total of 250 members in Central Norway. Invitation to the study was posted by BPM, who is a GP. A representative from each group contacted IBR by e-mail or phone to sign up for the study. Four CME groups were recruited through Facebook. Two other groups volunteered for the study after hearing about it through acquaintances.

### Participants

A total of 32 GPs from Central Norway participated in the study. All came from urban and semi-urban areas. Seven were males. Participants ranged in age from 34 to 62 years and had five to 26 years of experience as a GP. Of the participants, 28 were specialists in general practice and all worked in group practices. Most of the GPs performed cervical screening tests on a weekly basis, while 6 of the GPs, 4 of these being male GPs, performed cervical screening tests monthly. The compositions of their patient population varied. Some had more younger patients while others had a higher proportion of patients over screening age. These GPs knew many of their patients well, and some had several members from the same family as their patients.

### Data collection

Five focus groups consisting of 4 to 11 participants were conducted during spring 2024. The location for the focus groups was a meeting room in connection with a gynecology clinic where the following talk on HPV was held. Each group was moderated by PhD student and nurse IBR. Either MS or HG, respectively a sociologist and a linguist, both with substantial experience with focus groups, participated as co-moderators and made field notes during the focus groups. At the beginning of each session, moderators presented themselves and their professional background to promote transparency. All participants filled in a short questionnaire with background information before each focus group. Two of the CME groups were merged for practical reasons, resulting in 11 participants in one focus group. Although not all participants knew each other beforehand, the presence of familiar individuals contributed to a trusted atmosphere, though it took slightly more time to include all participants in the discussion during the merged focus group. We used a semi-structured topic guide covering the topics “GPs’ experience with the implementation of primary HPV screening”, “GPs’ assessment of patients’ health literacy and implications for practice” and “GPs’ practices for communication of abnormal cervical screening results”. Each focus group lasted between 60 and 90 min. All focus groups were audio recorded and converted into written text using the software “Speech to text”, then checked and validated by IBR.

### Data analysis

We analyzed the data material using Braun & Clarks reflexive thematic analysis (RTA), and applied a critical orientation to the analysis, questioning and engaging with the data beyond description [30]. The data analysis was performed by the author group, a multidisciplinary group with a nurse, a GP, a gynecologist, a sociologist and a linguist, all female. Individually, each member of the project group familiarized with the data through reading transcripts. All authors contributed to the second step of RTA through coding extracts from the transcripts, while IBR coded all data. As step three, IBR categorized the codes into preliminary themes, using mind mapping technique to help organize the codes. The authors identified and agreed on initial themes such as “How GPs share responsibility with their patients”, “GPs’ struggle with keeping updated”, and “use of professional judgement”. However, RTA is not as linear as the five steps suggest [30]. We constructed three final themes through going back and forth, reviewing and redeveloping themes. Concepts from theories on professionalism were used to sensitise the analysis to how professional roles and norms are shaped and expressed in clinical practice. In the analysis of data, a GP (BPM) and a gynaecologist (IB) contributed with clinal perspectives. We reflected on our own positions and presumptions through discussions between the authors. The interdisciplinary research group discussed interpretations, challenging insider and outsider views among the group throughout the research process. Not being medical doctors and native to the field allowed some of the members of the research group to approach the analysis with distance and perspectives from outside medicine.

### Ethics

The study did not fall under the remit of the Regional Committees for Medical and Health Research Ethics (REK) and therefore did not require their approval. The project’s plan for managing personal data was approved by Sikt - the Norwegian Agency for Shared Services in Education and Research (project # 914745). We followed the National Research Ethics Committees guidelines for research ethics in the social sciences and humanities [38]. Information about the study was sent to the participants per email prior to each focus group. Information was repeated and informed consent was collected from each participant at the beginning of each focus group session. Participants were informed both in writing and orally that participation was voluntary and how they could withdraw from the study. They were also informed that their confidentiality was ensured throughout all stages of the study and publication of the results. Data was stored in NICE-1, secured by two-factor authentication. During transcription, participants’ names were replaced by codes to maintain confidentiality. As support for reporting our study, we followed the Consolidated Criteria for Reporting Qualitative Studies (COREQ) [39].

## Results

Through our analysis, we identified three themes: “Meeting patients’ informational needs through expertise”, “Sharing responsibility for follow-up in HPV screening” and “Using professional judgement to adapt information”.

### Meeting patients’ informational needs through expertise

Despite having years of experience with cervical cancer screening, the GPs had faced difficulties keeping updated when Cervical Screen Norway changed their recommendations following the implementation of primary HPV screening. They could not remember having received information from the screening programme about new guidelines and discussed whether such information could have passed unnoticed during their busy workdays. As illustrated by this GP, revised routines were often first brought to their attention by patients.

I heard about this through patients who had questions. So, I started looking into and reading about it. I didn’t experience being presented with “here are new guidelines, here’s what you have to deal with”. I can’t remember getting it through any channel. But maybe it drowned in other information. There were no clear goals and changes in the guidelines. (GP1, FG1)

Being confronted with a variety of questions from the patients and with patients’ knowledge which often came from social media and friends was an opportunity to initiate dialogue, learn more about what the patients knew and correct misunderstanding. But it was also a challenge to their expectations towards themselves as medical experts, as this GP explains:

It’s always nice to know more than the patient. When they come and they know something that we don’t. For me, at least, it’s the feeling when they come and say, “that’s how it is.” Then you must pretend that you know it too. (GP3, FG2)

The GPs often used standardized texts from Cervical Screen Norway, or texts developed within their own clinic. These texts were adapted based on the GPs’ experience and knowledge of their patients to meet individual needs. As illustrated by one of the participants, the GPs were generally satisfied with the information they provided and designed their communication with the assumption that their patients could seek out and understand additional information. About this, one participant said:

I think that I provide sufficient information. If I had been the one receiving the letter, I would have been quite satisfied with it. I think it is exhaustive in a way. So, I think they don’t really need to seek more information. But otherwise, as you say, social media, the importance of taking tests and such, I believe they get a lot of input on that. But I think that my letter is more than good enough information for most of the patients. (GP3, FG2)

Not receiving any follow-up questions after opening for contact was interpreted by the GPs as a sign that the patients were well-informed. This reinforced their decision to continue their usual information practice. Patients’ access to other information sources was a likely explanation for why they were seldom contacted by their patients. For the GPs, this supported their perception that they were providing sufficient and evidence-based information in line with information from other official sources, such as the screening programme.

### Sharing responsibility for follow-up in HPV screening

Being knowledgeable about all aspects of the screening programme was experienced as unattainable for the GPs, and they needed to share responsibility for providing information and securing follow-up with the other actors in the programme. Struggling with keeping updated, receiving recommendations for follow-up from the laboratory was seen as a contribution to patient safety. As one of the GPs explained, this also simplified the GPs’ workload when communicating abnormal test results.

Because there are so many test results. There are codes, there are abbreviations, and there are different degrees of cell changes. And if we were to sit there and… then I think we would have taken an incredibly big risk. You have a busy day and check “what was this really, and what was the guideline really?” If you should do that, then you would have to make a checklist. But the fact that you get a recommendation for each case about what to do, then it can’t be missed, unless you don’t read. (GP8, FG4)

The GPs performed their tasks based on what they considered an appropriate sharing of responsibility. For their role in the screening program to be manageable, the GPs set boundaries for what they could be responsible for.

I think my responsibility must stop at some point. I think that ultimately the responsibility must lie with the patient. I can’t take responsibility for taking care of things for them. But there are a few patients where you think that “I’ll set up that check”, “I’ll send them a message”, “I’ll set up a check now and then”, “contact me in seven months”. (GP2, FG1)

For the GPs, sharing responsibility for follow-up was a tacit expectation, but was also expressed directly to the patients. This was done by explaining to their patients how responsibility is shared between the different actors in the programme to ensure correct follow-up. Test results were communicated with the knowledge that the screening program is organized to provide patient safety. Nevertheless, the GPs expressed uncertainty about how this was managed in practice by Cervical Screen Norway and felt the need to keep an eye on this, as explained by this GP:

But I also think that the Cervical Cancer Programme picks it up. And if there are patients who haven’t followed up, they get a message from them. But when is that? If you are supposed to take a test after 6-12 months and when a year has passed, they send out a message? (GP4, FG2)

What the GPs expected from their patients was explicitly communicated, and following up an abnormal test result was presented to the patients as an indisputable task. One of the GPs described how he explains this to his patients:

Explain to them that “fortunately we have the screening programme”. That’s kind of an intro. “Because these things take a long time [talking about the development of cell changes and cervical cancer]. We can follow up. You follow up. It is very important that you come back. That’s your part of the job.” (GP8, FG4)

Another way of securing follow-up was to inform the patients that they could expect both negative and positive test results being communicated from the GP and to follow up if they did not receive them within a certain time frame. Some of the GPs, however, informed patients only in the case of abnormal test results. Also, providing information about symptoms that should not be ignored was seen as critical. The GPs took responsibility for addressing what they considered to be information gaps in the standardized information letters, knowing that there is always a small risk that test results can be wrong or not always 100% reliable.

### Using professional judgement to adapt information

The GPs had different patient populations. Those with a high number of patients in the target group, had to prioritize how they spent their time when communicating the test results. For this, they used their professional judgement informed by former patient-experiences but mainly informed by knowing their patients and assuming these patients knew their GP as well. A long-term doctor-patient relationship was helpful for assessing patients’ information needs and whether it was necessary to give extra attention or support to secure follow up. This could involve providing extra reminders, reassurance, more thorough and detailed explanations, or proactively booking follow-up appointments for specific patients. The GPs’ experience that reciprocal GP-patient familiarity lowered the patients’ threshold for contacting their GP, had impact on their communication practices. This is illustrated by one of the participants:

I think most people know me well enough to contact me if they are wondering about something. I have experienced that earlier. I know that…Then it is almost not that important what I write. (…) If there is a patient I don’t meet so often, I would rather add a sentence saying, “just contact me if you have questions”. I am more direct about it if I’m not sure whether they dare to contact me. But most patients are not afraid to contact me. (GP3, FG2)

Knowing their patients gave the GPs opportunity to use their resources where needed the most. This familiarity also guided decisions about what information to give, how and how much, or if certain information should be held back.

So, I think I know exactly how to communicate to the different patients. Some need a lot, and some don’t really want the answer, as long as everything is okay. (GP5, FG2)

A long-lasting doctor-patient relationship provided knowledge about their patients’ health literacy. When not knowing their patients well, they assessed health literacy mainly by looking at the patients’ appearance and how they communicated during consultations. None of these GPs used any health literacy assessment tools but trusted their professional intuition.

It depends on that kind of feeling, or that kind of…that GP gut feeling rather than that… I don’t have a form. I don’t have something like; “now I’m going to assess your health literacy regarding Pap smears (…) But it’s, in a way, a more general thing that includes everything. (GP6, FG3)

Preventing anxiety was a central task for GPs when communicating abnormal test results. By knowing their patients, they could predict who would become anxious when receiving an abnormal test result. This prompted a more proactive and supportive approach. One GP explains this as follows:

It is about knowing the patient, so you know that the person will be completely put off by a letter and not being able to ask questions back to me immediately, then I call. (GP7, FG3)

Communicating the test results in a way that prevented anxiety and unnecessary worry, while at the same time stressing the importance of following up the test results was challenging. It was a main issue for the GPs to find the right balance between reassurance and awareness of the necessity of following up. This balancing act is described by one of the GPs:

But then you want them to be so worried that they come for follow-up tests (…) Isn’t that what we all struggle with? Try to put it that way. They don’t have to worry for a whole year but keep it in their attention, so they come for that check-up. (GP3, FG2)

Reassuring the patient implied both to stress that the GP was in control, using calming words and normalizing HPV. Combined with emphasizing the importance of the patient following up the test result, this information was experienced by the GPs to be more useful for the patients than explaining the test result and its implications in detail.

## Discussion

### Principal results

This study investigated how GPs communicate abnormal cervical screening results after the implementation of primary HPV screening in Norway. The key results from the analysis suggest that GP-patient communication is crucial in the cervical cancer screening programme. The professional expertise of the GPs includes communicative tasks, such as making the patient aware of the importance of follow-up without causing worry, communicating the patients′ own responsibilities, and using their professional judgement when assessing the patients’ health literacy. These GPs’ approach to communication was shaped by their struggle to keep updated on the new guidelines while addressing patients’ evolving knowledge and information needs. To meet these challenges, they shared responsibility for following up the test results both with the patients and the national screening programme. Sharing responsibility was guided by using their professional judgement, by knowing their patients and their needs, and by an underlying expectation that patients should take their part of the responsibility. Assuming varying levels of health literacy among their patients, GPs relied on their knowledge of each patient, as well as their professional judgment, when assessing the need for tailored communication.

### Strengths and limitations

A strength of the study is its design with using CMEs as participants in focus groups with GPs, which gave access to a group of study participants who are often difficult to recruit to research. Study participants who knew each other seemed to strengthen participants’ willingness to express opinions but could also have led to positioning as competent professionals. This was apparent during the focus group that merged two CME groups where not all participants were previously acquainted. During the merged focus group, participants were more careful in terms of sharing experiences and opinions.

A limitation of the study is the sampling strategy, where participants self-recruited based on topical interest. This limits the transferability of the results to GPs who have less interest in, or experience with cervical cancer screening. However, our data showed variance in professional approaches, which suggests sufficient information strength of the data. Moreover, study participants represented variation in gender, length of experience as a GP and in the number of patients in screening age on their GP lists. This contributed to data variation in experiences and rationales for their practices, but our study cannot claim to be representative for the full GP population in Norway. Merging two of the CME groups meant that some participants were unfamiliar with one another, which could explain why some contributed less in terms of sharing experiences and opinions. Participants were GPs from urban or semi-urban areas in one part of Norway with a relatively homogenous population. Results are therefore not directly transferable to GPs with more diverse patient populations, for instance immigrant patients with culturally diverse backgrounds. The GPs were not particularly familiar with the concept of health literacy, which led to vague discussions about this topic.

The multidisciplinary research group provided a variety of perspectives to the analysis, strengthening its validity. Moderators that were outsiders to the participants under study gave us extended insight into the participants experiences since the participants strived to explain internal concepts to us if we did not understand.

### Results in comparison with existing literature

#### Meeting patients’ informational needs through expertise

Norwegian GPs must deal with a growing number of clinical guidelines and demands [40], and staying updated on these is difficult [41]. In a study prior to implementation of primary HPV screening in Norway, GPs described the new flowchart for follow up of HPV tests as complex and challenging to understand [8]. GPs’ lack of information about HPV has made conversations with patients about HPV difficult [11]. The GPs in our study strived with acquiring knowledge on the field of cervical screening, challenging both their position as experts and meeting their patients’ informational needs. This is in line with experiences following the implementation of primary HPV screening in Australia, where GPs reported problems with understanding new routines, in turn creating challenges in explaining implications of test results to the women [9]. The paradigm shift from a paternalistic towards a doctor-patient relationships of mutual collaboration [28] has contributed to the involvement of the patient as a well-integrated part of GP’s professional practice [27]. But well-integrated in their profession is also the expectation to be in possession of and control of expertise which patients are dependent on [25]. Part of physicians’ knowledge comes from clinical experience [25]. In our study, not receiving questions from patients was an experience that justified current communication practices for the GPs. Balancing the expectation of being the expert on one side and expecting their patients to be active information seekers on the other side, was challenged when patients brought knowledge about HPV, mainly from the internet, into consultations. In their review looking at how physicians perceived it when patients brought information from the internet, Lu and Schulz [42] found that some physicians experienced this to undermine their medical authority, while others saw this as positive for empowering the patients and causing more efficient consultations. This variance was also visible in our results, where some GPs experienced being confronted with patients’ knowledge as an opportunity for dialogue about HPV.

#### Sharing responsibility for follow-up in HPV screening

Being in possession of expertise is the basis for professional autonomy, but also for the responsibility to choose proper actions [25]. Not being able to be fully updated on cervical screening and striving for balance in their workday, GPs in our study needed to share responsibility for follow-up with the other stakeholders in screening: the screening program and the women, viewing their patients as responsible and autonomous. This reflects the gradual, historical change from a paternalistic towards a mutual doctor-patient relationship [28], and its consequences for professional practice. Involving patients and encouraging their autonomy is a central skill in guidelines for physician education and practice [27]. In their study, Björk et al. [43]found that GPs viewed responsibility as something to be both given and taken within a reciprocal doctor-patient relationship, and they considered it an important task to communicate their expectations on shared responsibility. In the present study, responsibility was passed on to patients in person. Patients taking responsibility was also a tacit, general expectation guiding the GPs communication practice. In a study looking into GPs following up patients for suspected cancer, GPs transferred responsibility after assessing the patients’ capability to take it on, or after providing sufficient information and guidance. Some patients accepted being given this responsibility, while others needed to understand the rationale for follow up before they were willing to take it [44]. Available information is not always in line with what the patients need [17] and available information can be relevant for decisions about cervical cancer screening [45]. Hence relying too much on the patient’s ability to take responsibility for seeking information and following up may not always be adequate.

#### Using professional judgement to adapt information

When guidelines are missing or not comprehensive, physicians must rely on their professional judgement when solving their tasks [26]. One of the main results in our study was that the GPs tailored information guided by knowing their patients and the assumption that this knowledge was mutual. At the core of the GPs professional practice is acquiring knowledge about patients from interaction over time [46]. In our study, this was salient in the GPs’ discretionary reasoning when assessing their patients’ health literacy, primarily informed by knowing the patient. GPs in our study also used their gut feeling or former experience from interaction with patients in general when not knowing their patients well, which is in line with physicians building expertise through interaction with patients from experience [47]. Not using assessment tools for health literacy but trusting their intuition and knowing their patients over time has also been identified as a strategy among GPs in previous research [23]. Subjectively assessing health literacy is more accurate when the doctors know their patients better due to a relationship of a certain longevity [24]. The lack of consensus on appropriate tools for assessing health literacy makes systematic evaluation of health literacy difficult [48], which will be an obstacle to systematic implementation and use in GP practices. Knowing that the levels of health literacy in the population range widely [19], assessment tools could be helpful for equal evaluation of the patients. In our study, GPs interpreted lack of patient-initiated contact as a confirmation that their communication was successful, which can enforce their assumption of the success of both their discretionary reasoning and their intuition. But previous research shows that many patients are left with several unanswered questions about HPV after their GPs visit and often search the internet for answers [18].

Being able to understand their patients’ needs is a core professional competency for GPs, and the doctor-patient relationship enables this competency [46]. This can explain why the GPs in our study experienced knowing their patients as important for predicting who would become anxious when receiving an abnormal test result. They adapted their communication accordingly, which is in line with what gynaecologists in Sweden did to prevent anxiety from an abnormal test result [13]. Providing reassuring written information to most patients and promoting dialogue as a reassurance strategy to better meet the patients’ emotions and mitigate worry is found to be an important part of GP practice in previous studies internationally [49]. But the GPs in our study also experienced a reassuring and calming content to be important, hence adding this information to most patients. This was prioritized over a more detailed explanation of the test results. In cervical cancer screening, reassuring patients that their test results did not indicate cancer is a main concern [14,50]. However, being told to stay calm when receiving an abnormal test result can be experienced as dismissive [17], especially when patients lack sufficient knowledge about HPV [51]. Women who are unaware of HPV being a common infection is more likely to become anxious than those with more knowledge when testing positive for HPV [52]. The likelihood of patients following up screening programs can be increased by dialogue, as it opens for asking questions and being met emotionally [53].

Thus, the results suggest that follow-up of cervical screening is well suited to general practice, where GPs are in position to assess and respond to patients’ individual needs for information following an abnormal HPV test.

## Implications for practice

Although Cervical Screen Norway is developing information materials, communication between the screening program and GPs could be improved, as suggested by the results of the current study. This includes a common platform between the screening program and healthcare personnel to communicate changes in the screening program and provide guidelines for follow-up and how to communicate abnormal screening test results. Professional practices are shaped and influenced by the changing society not only in Norway, but worldwide. Hence, discussing our results from theories of professionalism, the results are useful for screening programs and GPs in other countries. Strengthening the collaboration between national cervical cancer screening programs and GPs has the potential to enhance the quality of screening programs through increased professional confidence. As a result, GPs can provide improved quality of patient information.

## Conclusion

When stakeholders are not sufficiently involved in changes within health services such as screening programs, maintaining their expert position and transferring sufficient information to patients are at stake. For GPs, it is important to receive enough information about cervical cancer screening, testing, and follow-up, to be able to safeguard patients with abnormal findings.

The results of our study highlight the need for improved information flow from Cervical Screen Norway to GPs when cervical cancer screening guidelines are changing, including support on how to communicate abnormal test results to the patients. GPs demonstrated strong professional commitment and a sense of responsibility in safeguarding patient care. To fulfil their role as trusted professionals, they must feel confident and well-informed – only then can they provide clear guidance and reassurance to their patients.

## Supplementary Material

Textbox_1_manuscript.docx
