# Supplementary material for: Communicating abnormal cervical cancer screening results – a focus group study with general practitioners in Norway
Source: Scand J Prim Health Care. 2025 Dec 5;44(1):1–13. doi: 10.1080/02813432.2025.2597785 (PMC12928665; doi:10.1080/02813432.2025.2597785)
Supplement: Textbox_1_manuscript.docx [file IPRI_A_2597785_SM0947.docx]

**Textbox 1:** HPV screening timeline during the study period following the national guidelines for cervical cancer screening in Norway

**Primary screening: HPV test every 5 years (women aged 25–69)**

**Test result determines the follow-up pathways:**

1. **HPV-positive test** → Cytology automatically performed at the laboratory
2. **HPV16** → Colposcopy & biopsy (regardless of cytology result) (gynecologist)
3. **HPV18**
   1. Normal cytology → Re-test in 12 months (GP*)
   2. Abnormal cytology → Colposcopy & biopsy (gynecologist)
4. **Other HPV types**
   1. Normal cytology → Re-test in 24/36 months (depending on age) (GP)
   2. Low-grade cytology → Re-test in 12 months (GP)
   3. High-grade cytology → Colposcopy & biopsy (gynecologist)
5. **Biopsy results**
   1. CIN1 → Follow-up in 6 or 12 months (GP or gynecologist)
   2. CIN2/3 → Excisional treatment (gynecologist)
      1. *CIN2 may be monitored in young women if criteria are met*

**GP- general practitioner*
